# “They call me the ‘Great Queen’”: implementing the *Malkia Klabu* program to improve access to HIV self-testing and contraception for adolescent girls and young women in Tanzania

**DOI:** 10.1186/s12978-024-01744-x

**Published:** 2024-02-07

**Authors:** Rachel Willard-Grace, F. Abigail Cabrera, Camilla Bykhovsky, Kayla Douglas, Lauren A. Hunter, Agatha Mnyippembe, Kassim Hassan Mgunya, Sandra I. McCoy, Jenny X. Liu

**Affiliations:** 1 Center for Excellence in Primary Care, University of California, San Francisco, San Francisco, CA USA; 2 School of Public Health, University of California, Berkeley, Berkeley, CA USA; 3 Health for a Prosperous Nation, Dar Es Salaam, Tanzania; 4 Institute for Health and Aging, Bixby Center for Global Reproductive Health, University of California, San Francisco, San Francisco, CA USA

**Keywords:** HIV self-testing, Drug shops, Adolescent health, Contraceptive methods, Private sector distribution, Tanzania

## Abstract

**Background:**

Adolescent girls and young woman (AGYW) comprise a significant proportion of new HIV infections and unintended pregnancies in sub-Saharan Africa yet face many barriers to accessing family planning and reproductive health (FPRH) information and services. Developed via human-centered design, the *Malkia Klabu* (“Queen Club”) program aimed to facilitate access to HIV self-testing (HIVST) and FPRH information and products at privately-owned drug shops. We sought to understand barriers and facilitators to program implementation in a 4-month pilot in Tanzania.

**Methods:**

Forty semi-structured interviews were conducted with participants in a cluster randomized trial of the *Malkia Klabu* program from November 2019 through March 2020, including 11 with AGYW, 26 with drug shopkeepers, and three with counselors at health facilities to whom AGYW were referred. Interviews were audio-recorded, transcribed, coded, and analyzed to identify key themes. The Consolidated Framework for Implementation Research (CFIR) was used to assess barriers and facilitators to program implementation at multiple levels. CFIR considers the outer setting (e.g., culture and systemic conditions), the inner setting where the intervention is implemented (e.g., incentives, relationships, and available resources), the individuals involved, the innovation as it relates to stakeholder needs, and the implementation process.

**Results:**

The *Malkia Klabu* program reshaped and directed the role of drug shopkeepers as providers of information and resources rather than FPRH gatekeepers. Key implementation facilitators included the program’s adaptability to a wide range of needs and stages of readiness among AGYW, ability to capitalize on AGYW social networks for driving membership, responsiveness to AGYW’s need for privacy, and positive contributions to the income and community standing of drug shopkeepers. Components such as HIVST were highly acceptable to both AGYW and shopkeepers, and the introduction of the loyalty program and HIVST kits in shops opened doors to the provision of FPRH products and information, which was further facilitated by program tools such as videos, product displays, and symbol cards. Although some shopkeepers maintained beliefs that certain contraceptive methods were inappropriate for AGYW, most appeared to provide the products as part of the program.

**Conclusions:**

The *Malkia Klabu* intervention's success was due in part to its ability to address key motivations of both AGYW and drug shopkeepers, such as maintaining privacy and increasing access to FPRH products for AGYW and increasing business for shops. Better understanding these implementation barriers and facilitators can inform the program’s future adaptation and scale-up**.**

*Trial registration*: clinicaltrials.gov #NCT04045912.

**Supplementary Information:**

The online version contains supplementary material available at 10.1186/s12978-024-01744-x.

## Abstract (plain language summary)

Adolescent girls and young women (AGYW) in sub-Saharan Africa have limited access to family planning and reproductive health products and information even though they are at greater risk of pregnancy and HIV infection. The *Malkia Klabu* intervention was designed with AGYW and shopkeepers from private drug shops to facilitate access to products and information through a loyalty program that included free products, prizes for purchases, educational videos, and a non-verbal system of requesting products through symbols. Qualitive interviews with AGYW, drug shop staff, and health system counselors suggested that the program helped provide greater privacy and confidence to AGYW while bringing new business to drug shops. These findings can help as the study team charts a pathway for scaling up the intervention.

**Supplementary Information:**

The online version contains supplementary material available at 10.1186/s12978-024-01744-x.

## Background

Despite being at high risk for both HIV acquisition and unintended pregnancy [1], adolescent girls and young women (AGYW) in sub-Saharan Africa face substantial cultural stigma and adult interference to accessing family planning and reproductive health (FPRH) services, such as HIV testing, contraceptive care, and pregnancy testing [2]. In Tanzania, 30% of people living with HIV are unaware of their status [3], and 46% of AGYW have never tested [4]. Self-care innovations, such as HIV self-testing (HIVST), may empower young women to know their HIV status and reduce reliance on facility-based testing. While there are government efforts to roll out HIVST more broadly in some countries, including Tanzania, little is known about effective strategies to ensure access and support for young people.

Drug shops are a potentially underutilized resource for linking young women to FPRH products. In Tanzania, privately-owned drug shops licensed as Accredited Drug Dispensing Outlets (ADDOs) are ubiquitous and easily accessible, with about 13,000 across Tanzania. Cistinct from pharmacies that are approved to sell controlled substances, ADDOs are approved to sell over-the-counter medications and some diagnostics (e.g., urine pregnancy tests) and are frequently women’s first point of contact for contraception, pregnancy tests, and informal counseling and referral [5]. Yet, at the same time, ADDO owners and dispensers may act as gatekeepers, interrogating AGYW customers who ask for FPRH information and products or denying these requests, in keeping with social norms that stigmatize adolescent sexuality [2].

Our study sought to develop AGYW-friendly FPRH services at ADDOs in the Shinyanga Region of Tanzania. We used human-centered design (HCD), a methodology that engages end users, in this case AGYW and drug shopkeepers, in the understanding of core problems and rapid design and prototyping of innovative solutions. Through a previously described process [2], we developed the *Malkia Klabu* (“Queen Club”) program to encourage AGYW to obtain and correctly use oral HIVST kits and other over-the-counter FPRH products (e.g., pregnancy tests, condoms, contraceptive pills), along with health facility referrals from ADDOs for confirmatory HIV testing and treatment or facility-based contraceptive methods (e.g., injectables, implants). The *Malkia Klabu* program was comprised of several components: a loyalty program in which eligible AGYW received a loyalty card through shopkeepers that enabled them to earn prizes through repeat shop purchases; free HIVST kits (as an opt-out sign-up gift for new members) and FPRH products, which could be accessed at any time by non-verbally pointing at discreet symbols on the back of the loyalty card; a FPRH sample product display for hands-on learning; and a tablet with videos to convey accurate information about HIVST and FPRH. The program relied on the social networks for AGYW for dissemination to avoid unwanted scrutiny from adults, as AGYW told their peers about the program and introduced them to ADDOs. As part of a four-month cluster randomized trial, intervention shops received training and materials from the study team to implement the *Malkia Klabu* program, including HIVST kits, prizes, FPRH sample product display, tablet, and processes for replenishing program commodities and getting reimbursed for other FPRH products (i.e., oral contraception, condoms, emergency contraception, pregnancy tests). Comparison shops received HIVST kits earmarked for free distribution to AGYW, but they did not receive other elements of the *Malkia Klabu* intervention. At the end of the trial, ADDOs implementing *Malkia Klabu* had higher AGYW patronage, distributed more HIVST kits and contraception to AGYW, and made more referrals for HIV and family planning services than comparison arm shops [6].

To inform future scaling of the *Malkia Klabu* program given the promising results of our pilot study, we conducted a qualitative study to understand the facilitators of and barriers to program implementation that shaped how the intervention affected the behaviors of participating ADDOs and AGYW. Guided by the Consolidated Framework for Implementation Research (CFIR) [7], we analyzed interviews conducted at the end of the trial but before release of study results with program participants, including AGYW, drug shopkeepers, and clinic-based referral counselors, to understand different stakeholders’ experiences with the pilot intervention. We sought to distill considerations for how *Malkia Klabu* might need to be adjusted, if at all, for achieving scale and the greatest impact on FPRH outcomes.

## Methods

The study protocol was pre-registered (clinicaltrials.gov: NCT04045912) and approved by the National Institute of Medical Research in Tanzania (reference NIMR/HQ/R.8a/Vol. IX/2862) and the Human Research Protection Program at the University of California, San Francisco (study 19-27888). The protocol and main outcomes for the study have been previously described [6]. For this qualitative study of program implementation, we conducted semi-structured in-depth interviews from late November 2019 (the final month of the trial) to March 2020 with AGYW, drug shopkeepers (owners and dispensers), and clinic-based referral counselors participating in the trial to better understand their experiences with the program.

### Setting and participants

Participants in the qualitative study were recruited from the 26 study sites in Shinyanga, Tanzania that participated in the larger cluster randomized trial, including 20 ADDOs randomly assigned 1:1 to the intervention or comparison arm, as well as six sites which were not part of the cluster randomized trial but which implemented the study protocol: three ADDOs that participated in the HCD program development phase implemented the *Malkia Klabu* intervention, as well as three non-ADDO pharmacies that participated in the comparison arm as part of field testing for potential program expansion. Eligible participants were members of the following three groups: (1) owners or dispensers at participating shops, (2) AGYW customers who completed exit surveys after shopping at a drug shop in either arm, and (3) clinic-based HIV counselors to whom participating drug shopkeepers referred AGYW customers in need of confirmatory HIV testing or other FPRH services. We aimed to recruit about 20 AGYW participants, 20 shopkeepers, and four referral counselors, with some flexibility given for achieving representation across AGYW (e.g., age, school enrollment, *Malkia Klabu* membership) and shop characteristics (e.g., size, performance, trial arm), and content saturation. There were six referral counselors engaged with the study across all sites, thus limiting the number of potential participants as counselors in the program.

### Interviews

Following an approved recruitment script, Tanzania-based researchers who were bilingual in English and Kiswahili approached shopkeepers from participating drug shops in person or by phone to invite them to take part in an interview. AGYW who had previously taken part in an exit survey when leaving a participating shop and also agreed to be contacted for an in-depth interview were then contacted by phone and invited to participate [8]. Eligible referral counselors were also contacted by phone to schedule an interview. Written informed consent was obtained at the time of the in-person interview; for AGYW under 18 years of age, the Institutional Review Board granted a waiver of parental consent. Three researchers trained in in-depth interviewing techniques conducted all interviews in Kiswahili following semi-structured interview guides (see Additional files 1, 2, 3); interviews lasted 30–60 min and were audio-recorded, transcribed verbatim, and translated to English. Interviews were concluded with each group when recruitment goals were met or when all potential interviews had been completed.

Shop owners and dispensers in both the intervention and comparison arms were asked about their experiences providing free HIVST kits to AGYW, which was newly introduced in all the shops (and the community) as part of the study. Intervention arm shopkeepers were also asked questions related to the adoption, fidelity, appropriateness, acceptability, and sustainability of the *Malkia Klabu* program. AGYW were asked about recent experiences visiting a drug shop, receiving and using an HIVST kit or contraception, and—if they were in the intervention arm – engaging with the *Malkia Klabu.* HIV counselors from nearby health facilities were asked about their experiences providing HIVST- and contraception-related counseling and referrals to AGYW who received their contact information at participating shops. Interview guides focused on sub-domains in the CFIR which were most appropriate for the pilot nature of the study, excluding domains better suited for adaptive learning when scaling programs. Participants received 10,000 Tsh (AGYW) or 15,000 Tsh (shopkeepers and referral counselors) in recognition of the time required for participation.

### Analyses

A four-member team not otherwise associated with the intervention design or program implementation conducted the analysis of the interview transcripts. We used CFIR [7] as a rubric for pre-specifying constructs for the structure of codes according to five major domains: intervention characteristics, outer setting, inner setting, characteristics of the individuals involved, and the process of implementation. We then identified emergent codes through an iterative process based on modified grounded theory [9, 10], in which the entire analysis team reviewed a sample of 11 transcripts and identified recurrent ideas, or “concept codes,” which were organized into the CFIR-informed codebook. The codebook was piloted and refined with six additional transcripts. Two team members used the codebook to conduct dual coding for each transcript using Dedoose [11]; they met to resolve discrepancies and refine definitions until consensus was reached. Coding and thematic development were conducted in an iterative process. The entire analysis team took part in thematic development, independently reading all text within a code to identify recurring ideas, comparing resulting themes across coders, and making within-group and between-group comparisons.

## Results

### Sample characteristics

Participants in the qualitative study included 11 of 16 AGYW customer exit survey participants who provided valid contact information for later interview follow-up (representing 5 shops, with a range of 1–6 interviews per shop), 26 of 31 invited drug shop owners and dispensers (representing 23 shops, with two interviews conducted at three shops), and 3 of 4 invited referral counselors. Primary reasons that AGYW did not participate included prohibitive school schedules (n = 3) and/or being physically remote and unable to meet the interviewers (n = 3). Primary reasons that shopkeepers did not take part was lack of time (n = 3) or being away from the shop for family reasons (n = 2). Half (13 of 26) of the participating shopkeepers and 10 of 11 AGYW (recruited across five shops) were part of the trial’s intervention arm offering the *Malkia Klabu*. Table 1 displays the characteristics of in-depth interview participants. All referral counselors and 19 of 26 (73%) of shopkeepers were women; about half of shopkeepers were owners.

**Table 1:** Characteristics of respondents

	AGYW (n = 11)	Drug shopkeepers (n = 26)	Counselors (n = 3)
Intervention arm, % (n)			
* Malkia Klabu* arm	91% (10)	50% (13)	–
Comparison arm	9% (1)	50% (13)	–
Gender, % (n)			
Male	0% (0)	27% (7)	0% (0)
Female	100% (11)	73% (19)	100% (3)
Age, mean (SD)	19.2 (3.3)	–	42.0 (13.7)
Has child	27% (3)	–	–
Student	55% (6)	–	–
Drug shop role, % (n)			
Owner	–	46% (12)	–
Dispenser	–	54% (14)	–

*AGYW* adolescent girls and young women, *SD* standard deviation

### Implementation facilitators and barriers

Overall, intervention shopkeepers and AGYW reported that the *Malkia Klabu* program was popular. AGYW reported purposely seeking out participating shops that accepted the loyalty card. In addition, shopkeepers actively recruited AGYW to join the *Malkia Klabu*, and most AGYW interviewees reported sharing information about the club with other AGYW by word of mouth. We identified several constructs related to each of the five CFIR domains that were salient to the implementation and uptake of the intervention.

#### Characteristics of the intervention

Many characteristics of the multi-faceted *Malkia Klabu* program were perceived by stakeholders as conferring advantages over the status quo, being adaptable to the needs of shopkeepers and AGYW alike, and being relatively simple to implement, which together facilitated implementation.

##### Benefits to shopkeepers’ businesses

Shopkeepers mentioned a variety of specific advantages to hosting the *Malkia Klabu* program. First, the *Malkia Klabu* program was a way to both attract business and increase their standing in the community. Several observed increased patronage by AGYW. As one shopkeeper shared:

*Yes, we have benefit a lot because those cards also advertise our business, in that part of buying we... explain to them that you have to buy medicine so that you can get gift so we have benefit through that.*

*(Dispenser, ADDO 28, offering Malkia Klabu)*

When asked about how others in the community perceived the shop’s involvement in offering the *Malkia Klabu*, one shop owner described positive community reception to the program and awareness of program components such as provision of menstrual pads. They also described the utility of the symbol card as a tool to discreetly provide products associated with social opprobrium:

*To tell the truth, they liked it, even though I didn’t know if they knew about the things at the back of the card. When a girl comes in and gets a pad, they like it… they see it as something that is good and helps out society, even though before I didn’t know if they knew anything about it.*

*(Owner, ADDO 7, offering Malkia Klabu)*

Overall, shopkeepers in the intervention arm expressed interest in continuing to participate in the *Malkia Klabu* program given the intervention also benefited their income. As one stated:

*It benefits me personally. Why not keep it? I would be very happy with myself if I would continue to have clients or young girls here at the store so that I could make a living [and] I would continue to provide services.*

*(Owner, ADDO 4, offering Malkia Klabu)*

##### Privacy advantages of HIVST

For AGYW participants, HIVST, which was newly provided by shops in both study arms, was popular because it offered privacy, was not painful, and returned quick results. Moreover, HIVST didn’t require any assistance from other people, which reduced concerns about inviting suspicion from other people regarding the reason for testing. As one AGYW *Malkia Klabu* member stated:

*Participant: I did it myself secretly, since she told me to do it a half an hour before eating or after, so I woke up at 5 am while the others were sleeping to go to do it… I woke up and [went to the] sitting room. No one is waking up at 5 am.*

*Interviewer: Alright… Why didn’t you decide to involve another person like brother, sister, or mother?*

*Participant: I was afraid because if I found the results are negative [i.e., positive for HIV], it is difficult to tell a close person, and the way they could behave toward me if they realized I were like that…it is really very difficult.*

*(AGYW 104, 22 years old, member of Malkia Klabu)*

While HIVST was available at both intervention and comparison shops, all but one AGYW interview participant were from intervention shops, so comments on HIVST from AGYW at comparison sites were limited. Nonetheless, the privacy advantage of HIVST was also echoed by comparison shopkeepers. A dispenser at one shop observed the value of being able to test in private versus visiting a hospital, which could prompt community gossip, saying:

*[Customers] were happy, because many are afraid to go to the hospital. Because they know when they go there, many [others] will discover that they have gone for an HIV test. So there are those who like to test on the streets.*

*(Dispenser, ADDO 10, comparison arm)*

The value of privacy was so compelling that it superseded concerns about cost for some AGYW. As one stated:

*Interviewer: If later you feel like you need to test again, and there are these tests available but [they] are sold [while] it’s done freely at the hospital, where would you choose to go?*

*Participant: I will buy this one.*

*Interviewer: Why while there’s that one at the hospital that might be testing for free?*

*Participant: Free? I will buy this one and test myself. It’s my secret, at home.*

*(AGYW 105, 24 years old, member of Malkia Klabu)*

Counselors also described the privacy advantage of HIVST. Coupling HIVST with telephonic counseling provided a mechanism to address additional questions without compromising the confidentiality afforded by the testing mechanism.

*Interviewer: Okay, do you think that making these test available within the community have been helpful in your work?*

*Participant: They are so helpful to the youngster, most of the youth don’t like to visit health facilities because here they meet with different people [like] parents, relatives and friends, so they don’t like but nowadays they are free because they even don’t know who do they speak to in the phone and they can ask any question.*

*(Counselor B)*

Other aspects of the HIVST kit that were valued by AGYW included a less painful test that did not require a blood sample and the ability to get quick results.

*Interviewer: Mmmh would you prefer such a test next time?*

*Participant: Yes… Because it’s not painful as a syringe and doesn’t take long to give results.*

*(AGYW 117, 17 years old, member of Malkia Klabu)*

##### Benefits to shopkeepers’ businesses

Shopkeepers mentioned a variety of specific advantages to hosting the *Malkia Klabu* program. First, the *Malkia Klabu* program was a way to both attract business and increase their standing in the community. As one shopkeeper shared:

*Interviewer: It seems like the girls became so familiar with you.*

*Participant: Absolutely, they became so familiar with me and they loved me so much, and until today they use to come to the shop, they call me “the great Queen” and I reply “the junior Queen.”*

*(Dispenser, ADDO 42, offering Malkia Klabu)*

Some noted the advantages of having and distributing HIVST kits, which conferred both social and professional standing to the shop, in comparison to other shops without HIVST.

*It is true that the status has changed. There [are] some other shops that did not get opportunity of having this self-test, and until today, if they came and found we have HIV self-test kit, people get surprised why do other shops don’t have it. So we have get status through this program.*

*(Dispenser, ADDO 28, offering Malkia Klabu)*

##### Tools facilitating access to FRPH

Tools such as the educational videos, displays, and symbol cards facilitated access to FPRH education and products at shops offering the *Malkia Klabu*. Both AGYW and shopkeepers reported use of the symbol card as a silent, convenient, and privacy-protecting tool to make requests, even if other customers were in the shop. As one dispenser described, the symbol card reduced the friction associated with having to verbalize a request and facilitated what was an otherwise fraught transaction for younger AGYW:

*Interviewer: And girls, when visited your shop, did they say aloud that they need condom or just point from the symbol card?*

*Participant: Some of them they just point, and some of them, we become friends. They were coming and say it aloud, “give me condom.” Some of them—specific of 15 years—most of them just point the product.*

*(Dispenser, ADDO 37, offering Malkia Klabu)*

Another shopkeeper described how AGYW who enrolled in *Malkia Klabu* because of the loyalty program’s prizes also used the FRPH videos to learn more about contraception:

*Malkia Klabu has made many girls be interested because of the gifts; many girls were registered because of the gifts. And also, this video that they were watching about family planning, it has helped them a lot… it helped us because when girls were watching it, they were understanding the benefits of family planning, and they keep using it.*

*(Dispenser, ADDO 28, offering Malkia Klabu)*

The display of FPRH products also allowed ADDO shopkeepers to share more about products available. As one AGYW explained:

*Interviewer: Did you get a chance to have a close look at those products, or did he just explain them and you left, or did you have another chance of looking or touching them?*

*Participant: He took me inside, and I looked at them one by one. He showed me all of those at the back.*

*Interviewer: And you had a chance of touching them?*

*Participant: Yes.*

*(AGYW 113, 15 years old, member of Malkia Klabu)*

The display even facilitated discussions more generally among shop’s customers, inviting curiosity among adults who may have similar FPRH needs:

*The display helped us a lot, it helped us to counsel even the adults who were not in the program. They used it to ask if those products were found at the shop and were telling them where to find those products which we did have.*

*(Owner, ADDO 36, offering Malkia Klabu)*

Thus, while the display was intended to attract AGYW, it was beneficial to the needs of a broader segment of customers and simultaneously helped to “market” the products sold by the shop.

In addition, the *Malkia Klabu* loyalty program and the provision of HIVST appears to have opened the door to conversations about FPRH products by establishing relationships between AGYW and individuals who could answer their questions. One counselor (B) observed, “You may find that if it is a girl and has tested for HIV and found to be negative, she will wish also to protect herself against pregnant, so you will find her asking you about family planning.” A shopkeeper echoed this thought, stating:

*[The Malkia Klabu] creates friendship and the girls do not fear to enter the pharmacy. Maybe she can tell you, “Can I have condoms?” They are used to me so they can easily ask for a P2 [emergency contraception] or PT [pregnancy test]. They are not afraid because they have built some sort of friendship.*

*(Dispenser, ADDO 21, offering Malkia Klabu)*

#### Navigating the inner and outer settings

Characteristics of the *Malkia Klabu* program aligned with the specific needs of AGYW clients and the ADDO shopkeepers (the inner settings) within the larger environment of pervasive stigma around AGYW sexuality (the outer setting), allowing them to provide and secure FPRH products within the confines of the larger culture.

##### Meeting the needs of AGYW

The *Malkia Klabu* program was designed to provide FPRH services despite the pervasive stigma associated with AGYW’s sexual behavior, a stigma affecting both “inner” and “outer settings” of the CFIR framework. Overcoming this obstacle required AGYW to be able to access FPRH products and information through accessible locations while maintaining privacy and avoiding public scrutiny [2]. Many AGYW participants expressed appreciation for being able to get FPRH products at more conveniently located drug shops rather than having to go to hospitals and meeting the basic need for accessibility:

*Interviewer: How do you see that issue of having something like that in the drug shop?*

*Participant: I think it is a good idea.*

*Interviewer: Why?*

*Participant: Because it facilitates a person to get those SRH [sexual and reproductive health] products when visiting the drug shop [rather] than going to the hospital.*

*(AGYW 116, 18 years old, member of Malkia Klabu)*

Key program components designed to address these needs of AGYW were the symbol card enabling nonverbal communication and informational materials (e.g., videos and product display), which participants reported were highly effective at introducing FPRH resources as AGYW joined the *Malkia Klabu*. One AGYW described the shopkeeper’s introduction to the loyalty program and the symbol card on the back, saying:

*He told me how to use it and explained me on how to use the symbols behind the card, he also added that in case I am in need of any product I just go to the shop and being given.*

*(AGYW 114, 15 years old, member of Malkia Klabu)*

Thus, the design of the loyalty cards immediately communicated to potential members the availability of FPRH products at the shop and how to access them in the initial interaction. Informational videos and displays of FPRH products further helped to provide education and correct widespread misinformation about contraception [2], and encouraged AGYW to ask additional questions. As one AGYW owner stated:

*Interviewer: You told me that [he] told you about the products he hangs at his shop. What was your take on that issue, seeing different products hanged on the pharmacy?*

*Participant: It was just good.*

*Interviewer: Why?*

*Participant: Because in case of unexpected sex one can go and take pills to protect pregnancy if she is a member of Malkia Klabu. Also, condoms can be used to combat various diseases.*

*Interviewer: Is there anything you disliked on the board that displays the products?*

*Participant: No.*

*(AGYW 117, 17 years old, member of Malkia Klabu)*

##### Leveraging AGYW networks and social communications

A major facilitator for the *Malkia Klabu* program was the use of existing social networks and relationships among AGYW to create awareness about the program. One design feature of the intervention was a deliberate decision to rely on AGYW’s peer networks, including leveraging the study’s Youth Advisory Board [2], rather than engage in more general market demand generation. This was intended to preserve the “exclusivity” of the program designed for AGYW, while not attracting unwanted attention to participants given prevailing social norms against adolescent sexuality. Nearly all AGYW participants reported sharing information about the club with their peers and recruiting others to join. As one AGYW reported:

*Interviewer: After joining the club, did you tell any other person apart from your mom?*

*Participant: Yes*

*Interviewer: Who else did you tell?*

*Participant: I told my neighbors called [name B] and another one [name C] on how it helps me… They said they will join, and I directed them the pharmacies, but I haven’t asked them if they’ve visited them or not…*

*Interviewer: Is there any other person that you’ve informed?*

*Participant: I told a good number of people at church… I told girls named [name D], [name E], and [name F].*

*(AGYW 117, 17 years old, member of Malkia Klabu)*

The importance and excitement of HIVST was a particular advantage of the *Malkia Klabu* program that participants were not only keen to share with their peers, but made additional efforts to counter negative influences, such as misinformation provided by adults in the lives of their peers:

*Interviewer: And that time did you tell other people about this test after you used it? Your friends, neighbors?*

*Participant: Yes.*

*Interviewer: Who did you tell?*

*Participant: I told three girls, they were along the road the first time we went to the pharmacy to take the tests, they told us their mother told them not to go there to take them, that you are told lies, they give you fake tests and when you actually test is when you get HIV.*

*Interviewer: Mmmhmmh. What did you tell them?*

*Participant: I told them your mother is lying, their mother forbidden them to go there, they used to escape from their mother and go, they took the tests and hid on a pit and tested, when they go to Malkia card when they are given money they kept it, when the time reach that they are asked to go somewhere, they also go to the pharmacy, and then came back home.*

*Interviewer: Mmmh, so they were in Malkia but their mother didn’t know they are in, and she didn’t know they got the test?*

*Participant: Eeeh.*

*Interviewer: Mmmh mmmh, is there anything they told you that they got from Malkia besides tests, is there any other products they told you they got it there?*

*Participant: Yes.*

*Interviewer: They said they got what else besides those prizes, items from the back of the card?*

*Participant: Those products from the pharmacy, they said they asked for a pregnancy test, and they were given.*

*(AGYW 113, 15 years old, member of Malkia Klabu)*

As demonstrated by this quote, exposure to the *Malkia Klabu* intervention not only resulted in provision of FPRH products to AGYW but also an increase of knowledge and confidence in sharing unbiased health information with peers.

Peer recruitment, in turn, brought business to the drug shop, expanding their customer base and further incentivizing shopkeepers’ participation in the program. Several AGYW mentioned that they specifically looked for the crown sticker placed in *Malkia Klabu* intervention shops when they were shopping, thus highlighting the role of the program in channeling business to participating shops.

##### Parental engagement varied widely

While *Malkia Klabu* was not designed for direct engagement from parents, AGYW participants noted that some parents were not only aware of the program, but that they were highly supportive of their daughters’ participation. In particular, AGYW tended to talk with mothers about HIVST results.

*Interviewer: What made you do the test?*

*Participant: I went there because I wanted to know my health status.*

*Interviewer: Mmmh!*

*Participant: Yes, because from the start of this year I had tested only once at [health facility A]. So when I heard that there are pharmacies that provide test equipment, I told my mom and she told me to go. I went and tested, and the results were good.*

(AGYW 117, 17 years old, member of Malkia Klabu)

Some parents even went with their daughter to thank the shopkeepers personally for hosting such a program.

*Participant: I like about testing, the kids now they know about their health status, that they need to pursue their dreams because they know their health, they thank their parents for safe birthing, so a lot were happy because of that. Most of them came with their parents.*

*Interviewer: What about those who came with their parents, what did they come for?*

*Participant: They came to thank me that their children know their health.*

*(Dispenser, ADDO 2, offering Malkia Klabu)*

#### Characteristics of individuals

##### Broad appeal to AGYW

Program adoption was facilitated by the *Malkia Kalbu’s* ability to both broadly appeal to AGYW as a loyalty program with prizes in addition to appealing to those with specific FPRH needs. AGYW with no interest or confidence in FPRH were attracted to the program through the prizes offered by the loyalty program. As one AGYW stated:

*Interviewer: Which thing attracted you most in the Malkia Klabu?*

*Participant: The gifts ha ha ha! (laughing)… because each month I must buy the pad, but now I get them free.*

*(AGYW 104, 22 years old, member of Malkia Klabu)*

AGYW did not describe the cost of products needed to get loyalty card purchases to be a barrier. Of note, all of the FPRH products were free to AGYW through the program, and the products that they had to purchase in order to earn points could cost any amount of money, so many purchased some paracetamol. The median purchase amount was 200 Tsh (US $0.09). Some AGYW participants were particularly engaged and motivated, and they sought creative ways to earn loyalty points. For example, some AGYW reported running errands to *Malkia Klabu* shops on behalf of family or neighbors in the face of their own limited financial means. As one AGYW described:

*Participant: Yes, money is also a challenge*

*Interviewer: Alright… so… what were you doing now to get the money, even the 200 shillings you told me?*

*Participant: When I hear someone says, “Somebody go to buy drug for me,” I become like, “Oh, please let me go and buy it for you”… ha ha ha!*

*Interviewer: Have you ever bought the drugs for your neighbor?*

*Participant: Oh yes, but she doesn’t know if I have a Malkia card. I just told her that let me go to buy it for you because I go there.*

*(AGYW 104, 22 years old, member of Malkia Klabu)*

Although the program required that AGYW make some type of purchase (no minimum specified), respondents did not indicate that this hindered their participation. Rather, AGYW participants indicated that they found ways to make purchases, including for others. There were no reports of AGYW resorting to risky behaviors (e.g., transactional sex) to attain prizes.

##### Empowering AGYW for FPRH

All AGYW interview participants reported having successfully HIV tested using an HIVST kit distributed by the program. HIVST was the sexual health activity most readily adopted by AGYW, whose primary motivation was to “know one’s status.” Knowing one’s status was equated with control and power. Confirming that they were HIV-negative was a relief (peace of mind), but AGYW and shopkeepers also expressed that finding out that one was HIV-positive would also enable them to take control of their health. As some AGYW participants explained:

*Interviewer: Why didn’t you wait for a week contemplating?*

*Participant: Because I intended to test, why should I wait for a whole week? I tested right away on reaching home, because you need to know your status.*

*(AGYW 102, 23 years old, member of Malkia Klabu)*

*Interviewer: What made you to take such a testing kit?*

*Participant: I was thinking that when I took this testing kit and make a check-up, I will be aware of my health status. If I found positive, I will start a dose for ARV [antiretroviral therapy], but if negative, I will continue to protect myself.*

*(AGYW 116, 18 years old, member of Malkia Klabu)*

AGYW reported that the videos describing HIVST and their subsequent discussions with shopkeepers bolstered their knowledge and self-efficacy and enabled them to successfully perform HIVST.

Because HIVST kits were provided at no cost through the program and had a high level of social acceptability, they provided a step toward greater knowledge and self-confidence in engaging in other FPRH services, even for AGYW reticent to engage in conversations about contraception. AGYW who joined the *Malkia Klabu* primarily for loyalty rewards reported receiving an HIVST kit, and subsequently accessing other program FRPH products. As one AGYW club member stated:

*That day when I went to buy the drug, I bought it and he told me to go and take a gift. He showed me an HIVST kit and said you can have it and go test for yourself whether at home or any place of your interest. I decided to take it and I tested myself in order to know my health status. And as the days went on, I went to take a pregnancy test; I also tested myself in order to know how I am.*

*(AGYW 104, 22 years old, member of Malkia Klabu)*

AGYW reported securing information and products for contraception through the program. Watching videos about contraception emboldened some AGYW to ask shopkeepers follow-up questions, and the symbol card facilitated requesting desired FPRH products. One AGYW commented on the program’s impact on confidence and self-efficacy, saying:

*Participant: Malkia Klabu also makes girls confident when they meet boys.*

*Interviewer: Mmmh! How does it make a girl confident?*

*Participant: For example, when one uses the birth control methods… It brings confidence as one becomes sure of protecting oneself from unplanned pregnancy even when you have sex.*

*(AGYW 117, 17 years old, member of Malkia Klabu)*

##### Transforming shopkeepers’ roles

Unlike HIVST which were newly introduced by the study, FPRH products were widely available at drug shops but were often perceived to be inaccessible due to the tendency of shopkeepers to act as gatekeepers for AGYW’s FPRH needs [2]. While the program was not designed to change the larger cultural norms stigmatizing young women’s sexuality, the design challenge necessitated steering shopkeepers away from acting as gatekeepers inhibiting access to FPRH supplies to resources, and toward acting as providers of health care who facilitate access. As such, the program appeared to have a transformative effect on the role of shopkeepers. AGYW participants described how shopkeepers helped them to access information and FPRH products.

*Interviewer: Alright… did you feel free to ask questions to the shop seller about the test kit?*

*Participant: Yes, I was free because she was telling us to be free to ask any questions.*

*(AGYW 111, 17 years old, member of Malkia Klabu)*

Shopkeepers themselves reported receiving follow-up questions about FPRH products and proactively offering assistance:

*Interviewer: What part of the Malkia program did the girls like most?*

*Participant: First the gifts, then the education on the right use of things like condoms. Some did not know how to use them, so I explained [it] to them first when they asked for them.*

*(Owner, ADDO 12, offering Malkia Klabu)*

However, some beliefs were more resistant to change. For example, unlike their embrace of HIVST kits and barrier contraception methods, some shopkeepers expressed negative views about AGYW’s use of hormonal methods. One said of hormonal birth control, “I think… they are harmful to them… I think these pills should be used by someone who already has babies” (Dispenser, ADDO 27, offering *Malkia Klabu*). Some held similarly negative views of emergency contraception. Concerns ranged from a belief that frequent use would result in adverse impacts to health or fertility to a belief that it might encourage greater sexual activity and increase the risk of HIV transmission. In most cases, these reservations did not prevent the shopkeepers from providing the FPRH products. For example, one shopkeeper described an encounter with an AGYW, saying:

*Participant: She came at the shop and shows me the card, and she said please can I have this she shows me the product that she wants at the back of the card.*

*Interviewer: What did she want?*

*Participant: P2 [emergency contraception].*

*Interviewer: What did you tell her?*

*Participant: I didn’t tell her anything because she came and show me. She didn’t say anything so I just give it to her.*

*(Dispenser, ADDO 28, offering Malkia Klabu)*

However, when asked immediately after what they would change about the program, the same shopkeeper stated:

*I think P2 [emergency contraception] should be removed completely. Girls, they don’t use condoms, they don’t even think that there is HIV. It more better for those girls who are coming with their cards to take condoms, according to me that is good instead of this who she is coming for P2.*

*(Dispenser, ADDO 28, offering Malkia Klabu)*

This pattern of voicing reservations about particular products, yet providing those same FPRH products was not uncommon among shopkeepers offering *Malkia Klabu*.

### Implementation process

Interviews explored the process of implementing *Malkia Klabu* from participating shopkeepers in the intervention arm of the pilot trial. Overall, intervention shopkeepers reported minimal challenges in implementing the intervention and expressed wishes that it continue.

*Interviewer: What has been the biggest challenge in continuing using this program?*

*Participant: No, haven’t seen any challenges.*

*Interviewer: Anything to help the program maybe?*

*Participant: Nothing.*

*Interviewer: Do you have any feedback relating the program?*

*Participant: If it’s possible to continue it should continue.*

*(Dispenser, ADDO 2, offering Malkia Klabu)*

ADDO shopkeepers observed that the videos and displays made it easy to provide information and invite conversation with AGYW.

*[The] tablet helped me much, you know. You talk to them, but in order to make it simple you put video for them to watch. If she is just one you give her earphone, she watch... It was helping me to start. It simplified, and they trusted.*

*(Dispenser, ADDO 37, offering Malkia Klabu)*

Similarly, counselors reported that receiving calls from AGYW after testing was not burdensome or difficult, in part because this counseling was aligned with their usual day-to-day activities. For example, Counselor A stated: “No, it did not give me difficulty because many were not just telephone calls... you call you give advice [and sometimes] you tell come to look at.”

Considering the different components of the intervention, shopkeepers were more engaged in implementing some components of the intervention than others. For example, shopkeepers reported minimal challenges to implementing the loyalty program and gift distribution.

*Interviewer: How did you feel to allow girls to take gifts from the mystery box?*

*Participant: I felt good because it is their right.*

*Interviewer: What kind of product did they like from the mystery box?*

*Participant: Sanitary pads.*

*Interviewer: Okay. Did you face any challenge from this program of ladies taking their gifts from their mystery box?*

*Participant: No.*

*(Dispenser, ADDO 2, offering Malkia Klabu)*

Components of the intervention that some shopkeepers reported being less frequently used included the video about FPRH methods and the product display, possibly due to cultural stigma around provision of FPRH information or products to AGYW. Some shopkeepers nonetheless took a more active approach to referring AGYW to the educational aids, such as the video or displays about FPRH products, when they felt the need arose. As one AGYW described of her recruitment into the program:

*In guidance and counselling, first of all, she found my results were positive [i.e., did NOT have HIV], so she insisted me to continue protecting myself by avoiding to have a sexual intercourse without using a condom. And when she finished, she told me don’t forget there is a card I told you that day you were in hurry… Then I took [the card]. She also brought me other FPRH products and showed me a video [what] they do. So she tempted me to join the club, and I joined it that day*

*(AGYW 107, 21 years old, member of Malkia Klabu)*

## Discussion

Developed through a human-centered design process, the *Malkia Klabu* program was intentionally designed to work within the confines of the existing cultural norms and institutional structures, leveraging the beneficial features of these environmental factors (e.g., convenient health product access through community drug shops) to work in concert toward improving access to HIV/FPRH services for AGYW [2]. Our analysis of qualitative data from our mixed-methods evaluation found that the intervention was successfully implemented and enthusiastically adopted by AGYW and drug shopkeepers participating in the pilot study. Notable contributors to the success of implementation included the program’s ability to provide wrap-around support to connect AGYW to FPRH information and products, its relative advantage in that it appealed to AGYW at varying stages of readiness and self-efficacy to engage with FPRH, and relative advantage in that it increased business for participating drug shops.

The multifaceted *Malkia Klabu* program was designed to respond directly to the core challenges that AGYW face in accessing HIV/FPRH services [2]. While the pilot study introduced HIVST, which in-itself is designed to empower preventive self-care, the enhanced delivery of HIVST alongside contraceptives in drug shops offering *Malkia Klabu* appeared to more holistically address AGYW’s needs and may be positioned to foster lasting behavior change through multiple motivational levers and tools. Product availability at community drug shops enabled AGYW to avoid potentially stigmatizing treatment at hospital clinics, and providing different opportunities for learning, engagement, and understanding of FPRH information and products within drug shops facilitated knowledge and uptake while also benefiting shops’ marketing. Few other interventions have specifically targeted the needs of AGYW who may not have children or be in long term relationships, and for whom the stigma of seeking HIV testing or FPRH services is highest; many previously described programs relied upon hospital or health centers, settings in which AGYW may experience heightened public scrutiny [2, 12, 13]. This study builds on promising results showing that ADDOs are a common first source for healthcare needs for people in Tanzania [5] and that they can expand access to testing and treatment for other conditions (e.g., malaria) [14]. ADDO-based programs like *Malkia Klabu* provide a unique opportunity to facilitate access to HIVST and FPRH services and create a welcoming environment for AGYW. AGYW participants in this study indicated that the program fit well into their daily lives. Some participants even found ways to take advantage of their family’s regular errands and make purchases for others to earn loyalty points, creating more opportunities to obtain preventive FPRH products and information before they are critically needed.

Researchers have previously observed the potential expand access to FPRH services through privately-owned drug shops [15]. However, the prevailing culture of stigma toward the sexuality of AGYW has posed challenges to providing AGYW-friendly services that ensure their privacy. With rates of HIV testing and use of FPRH products remaining low, and concerns about privacy, stigma, and access reported as some of the biggest barriers to greater use of health center-based services [16–18], there is an urgent need to develop creative points of access tailored to the unique needs of AGYW. This study demonstrates the potential of engaging drug shops as partners to expand access to HIVST and FPRH information and products to AGYW in places such as Tanzania and elsewhere in sub-Saharan Africa. ADDO shopkeepers interviewed for this study were receptive to an expansion of their role to serve as counselors and service providers to AGYW, provided that their businesses also benefited. They welcomed the influx of AGYW customers into their shop and noted the positive impacts of this expanded role on their standing in the community, including from some appreciative parents of AGYW.

This study has several limitations. AGYW and shopkeepers interviewed for this study were located in Shinyanga, Tanzania, and additional research is needed to understand how the *Malkia Klabu* intervention may need to be adapted as it is expanded to other settings, such as more rural areas or other countries. The small sample of AGYW interviewees was drawn from a larger pool of shop patrons to ensure variety in participant characteristics (e.g., in-school and out-of-school AGYW, AGYW with and without children), but they are not intended to be a representative sample. Most AGYW interviewees (6 of 11) were recruited from one ADDO, so their experiences may be overrepresented in this sample. Moreover, all but one AGYW interviewee were recruited from intervention shops, so the ability to comment on AGYW experiences at comparison shops is limited. Although interviewers sought to create an environment free of judgement, social desirability bias may have impacted responses. Both shopkeepers and AGYW may have been reluctant to speak negatively of the program due to having received products and services through it.

Adolescents and young woman in sub-Saharan Africa are among the most vulnerable populations for new HIV infections or undesired pregnancies. Addressing their need for private and AGYW-friendly services requires new approaches and partnerships. The *Malkia Klabu*, developed in partnership with AGYW and shopkeepers through an HCD process, is a promising intervention that has demonstrated several key advantages regarding implementation and scalability and may help to transform the role of shopkeepers to meet the family planning and reproductive health needs of AGYW.

### Supplementary Information

**Additional file 1**: In-Depth Interview Guide—ADDO Owners and Employees.

**Additional file 2**: In-Depth Interview Guide—Adolescent Girls and Young Women.

**Additional file 3**: In-Depth Interview Guide—Health Facility Staff.

## Data Availability

The datasets used and/or analyzed during the current study are available from the corresponding author on reasonable request.

## Abbreviations

ADDO: Accredited Drug Dispensing Outlet
AGYW: Adolescent girls and young woman
CFIR: Consolidated Framework for Implementation Research
FPRH: Family planning and reproductive health
HIVST: HIV self-testing
HCD: Human-centered design
