# Supplementary material for: “They call me the ‘Great Queen’”: implementing the Malkia Klabu program to improve access to HIV self-testing and contraception for adolescent girls and young women in Tanzania
Source: Reprod Health. 2024 Feb 7;21:21. doi: 10.1186/s12978-024-01744-x (PMC10848389; doi:10.1186/s12978-024-01744-x)
Supplement: Supplementary file 1 — Additional file 1: In-Depth Interview Guide—ADDO Owners and Employees. [file 12978_2024_1744_MOESM1_ESM.docx]

# In-Depth Interview Guide – ADDO Owners and Employees

_________________________________________________________________________

|  | Date: | **(DD/MM/YY)**: ____ /____ / ____ |
| --- | --- | --- |
|  | Interviewer name: |  |
|  | Participant ID: |  |
|  | Participant age: |  |
|  | Participant sex: |  |
|  | Participant role at ADDO: |  |
|  | ADDO ID: |  |
| 8. | Start time: |  |
| 9. | Finish time: |  |

**Introduction and consent**

1. ***Introduce yourself and the study.***
2. ***Obtain written informed consent [READ CONSENT FORM]***
3. Did you ask if the participant has any questions?  YES  NO
4. Did participant agree to participate?  YES

NO 🡪 *STOP*

1. Did you give participant a copy of consent?  YES  NO
2. ***Obtain permission to audio record.***

We would like to audio-record the interview so that we can record everything we discuss here today. You can also request that I turn off the recorder at any time.

1. Is it okay with you if I audio-record?  YES 🡪 *TURN ON RECORDER*

NO 🡪 *TAKE NOTES*

1. ***Turn on recorder and say Participant ID into the recorder!***

***Instructions: The questions below outline main categories of questioning. The sub bullets detail topics for further probing. There is no need to ask every question, and no need to stick to this order of questions, but rather to focus on the areas in which the respondent has the most to say.***

Before we start, I would like to remind you of some important things to keep in mind during our conversation. Please remember that everything about this study is completely voluntary, and you should not feel compelled to share anything you do not want to. Everything you say will be kept confidential; nothing will be shared with government officials, other people who work at your shop, or anyone outside of the research team. As we are asking questions that might lead you to think about sensitive topics, if you do not like a question, or if you want to end the interview, you are free to do so. Nothing bad will happen.

Some of the questions ask about customers in your shop. When you are answering these questions, please don’t tell me the customers’ names or identifying information about them. Some questions ask about adolescent girls and young women in your community. When I say “adolescent girls and young women” (or simply “girls”), I am talking about people around ages 15 to 24 years old.

**A. Introduction**

First, I’d like to learn a little bit about you and your time at this drug shop:

How long have you [**worked at / owned**] this shop?

What’s the best part about your work? What about it do you enjoy or makes you happy?

What’s the hardest part about your work?

**B. Beliefs about demand and opportunity to restock HIV self-test kits** *(For owners only)*

***Note: Section B should be completed in Qualtrics form and should be asked exactly as written.***

Now I would like to ask you a few questions about your experience selling the white-label HIV self-test kits.

1. How many white-label self-test kits do you currently have in stock? **(*Ask participant to count remaining kits in front of you, if possible, to verify the count yourself.*)**
2. Think back about your sales in December last year. Without looking back at the sales log, how many white-label self-test kits did you sell in the first week of December? At what price per kit (on average)?
3. How many white-label self-test kits did you sell over the whole of December? At what price per kit (on average)?
4. How many white-label self-test kits do you think other shops participating in this study sold on the first week of December? At what price per kit (on average)?
5. How many white-label self-test kits do you think other shops participating in this study sold over the whole month of December? At what price per kit (on average)?
6. Suppose you still had white-label HIV self-test kits in stock. How many white-label self-test kits do you think you could sell next week? At what price per kit (on average)?
7. How many white-label self-test kits do you think you could sell over the next month? At what price per kit (on average)?
8. Think about other shops participating in this study and suppose they still had white-label HIV self-test kits in stock. How many white-label self-test kits could they sell next week if they sold them at: ***(randomize choice order, ascending or descending)***
   1. 10,000 Tsh
   2. 5,000 Tsh
   3. 2,000 Tsh
   4. 0 Tsh (distributed for free)?

Now that you have experience selling self-test kits to the community over the past few months, we will give you the opportunity to purchase **5 additional self-test kits** to continue selling to the community. Note that there will be no additional pink label kits and *all* customers can now buy white label kits, including adolescent girls and young women. It is up to you to determine how much you want to sell the test kits for. This is the *only* time we will give you the opportunity to purchase additional self-test kits.

I would like you to tell me how much money you would be willing to spend to purchase 5 self-test kits. I will ask you about various amounts of money per test kit, and whether you would like to purchase 5 test kits at that amount per test kit. For one of the price levels, you will *actually* purchase the self-test kits or not purchase self-test kits at all, so *these choices matter and you should make sure to think carefully and give the most accurate answer*.

We will use a lottery to pick one of the shilling amounts you mentioned. We only have a limited number of self-test kits left so a lottery is a fair way to decide who gets to purchase the remaining test kits. The price you pick from the lottery could be different from the price picked at other shops, by chance.

Do you understand? ***(Answer any questions from respondent until they completely understand the instructions.)***

Let’s go through an example with this piece of chitenge to make sure you understand the process.

1. Would you like to purchase this piece of chitenge at a price of ____ Tsh? **(*Repeat for each price level*.)**

| a) | 1,000 Tsh | □ Yes | □ No |
| --- | --- | --- | --- |
| b) | 2,000 Tsh | □ Yes | □ No |
| c) | 3,000 Tsh | □ Yes | □ No |
| d) | 4,000 Tsh | □ Yes | □ No |
| e) | 5,000 Tsh | □ Yes | □ No |
| f) | 6,000 Tsh | □ Yes | □ No |

*Roll dice and record answer ____.* You rolled a *-insert number-*. This corresponds to a price of *-insert number-* Tsh.

- ***If respondent picked “Yes” at the price level****.* You previously said you would like to purchase this piece of chitenge at *-insert number-* Tsh. You will now purchase the chitenge at *-insert number-* Tsh.
- ***If respondent picked “No” at the price level.*** You previously said you would not want to purchase this piece of chitenge at *-insert number-* Tsh. You will not purchase the chitenge at *-insert number-* Tsh, or at all.

Now I will ask you how much money you would be willing to spend to purchase 5 test kits. If you purchase the test kits, we will complete the transaction right after the lottery.

1. Would you like to purchase *5 self-test kits* at a price of ____ Tsh per kit? You will have to pay ____ Tsh in total **(*Repeat for each price level.)***

|  | **Price per kit** | **Total payment** | **Purchase?** | |
| --- | --- | --- | --- | --- |
| a) | 1,000 Tsh | 5,000 Tsh | □ Yes | □ No |
| b) | 2,000 Tsh | 10,000 Tsh | □ Yes | □ No |
| c) | 3,000 Tsh | 15,000 Tsh | □ Yes | □ No |
| d) | 4,000 Tsh | 20,000 Tsh | □ Yes | □ No |
| e) | 5,000 Tsh | 25,000 Tsh | □ Yes | □ No |
| f) | 6,000 Tsh | 30,000 Tsh | □ Yes | □ No |

*Roll dice and record answer ____.* You rolled a *-insert number-*. This corresponds to a price of *-insert number-* Tsh per test kit.

- ***If respondent picked “Yes” at the price level****.* You previously said you would like to purchase 5 self-test kits at *-insert number-* Tsh per test kit. You will now purchase the 5 self-test kits for *-insert number-* Tsh in total.
- ***If respondent picked “No” at the price level.*** You previously said you would not want to purchase 5 self-test kits at *-insert number-* Tsh per test kit. You will not purchase the self-test kits at *-insert number-* Tsh in total, or at all.

***(Complete transaction if respondent picked “Yes” at the price level.)***

**C. HIV self-testing**

Now I will ask you about your experiences providing HIV self-test kits (HIVST) at this shop.

| **Topic** | **Main question** |
| --- | --- |
| Adoption | Have you been providing HIVST to customers at your shop? Why or why not? (***Probe about adolescent girls and young women specifically.***) |
| Fidelity | Please describe your procedures for selling HIVST to a customer. Can you tell me about the last time you sold HIVST to a customer? (***Probe about AGYW*.**)   - How do customers learn that your shop is offering HIVST? - How do customers receive information about how to use HIVST? - ***Intervention ADDOs only*:** Can you tell me about the last time a customer watched the HIVST video in your shop? (***Probe about AGYW*.**) Have you experienced any challenges with this video?   You may have counseled customers about how to use or interpret HIVST while working at the shop. Can you tell me about the last time you counseled a customer about HIVST? (***Probe about AGYW*.**)   - Tell me about your general process when you counsel customers about HIVST. - What works well while counseling customers about HIVST? What works less well? Can you give me a specific example   Can you tell me about the last time a customer came in to ask you about positive or unclear HIVST results? What steps did you take to encourage him/her to seek confirmatory testing at a health facility?  Can you tell me about the last time a customer asked a question about HIVST that you did not know the answer to? Are there areas where you would like additional training on offering HIVST at your shop?  ***ADDO owners only*:** How confident are you that your staff have been offering HIVST as it was intended to be offered? |
| Acceptability | Do you think that providing HIVST at your shop has been helpful to you in doing your job, or has it made your job harder? Why? (***Probe for specific examples.***)   - What do you like about it? Dislike about it? - What has been easy about it? Difficult about it?   Has offering HIVST helped you serve your customers better? Why or why not? (***Probe about AGYW.***)   - ***If yes:*** Can you tell me about a time that your customer benefited from your shop offering HIVST?   How do your colleagues generally feel about offering HIVST? What kinds of things do they say about this?  How has offering HIVST has affected business in the shop? Why do you think this is?   - Have you experienced any changes in how many or the types of customers who come to your shop? (***Probe about AGYW.***) Why do you think this is?   How has offering HIVST affected the shop’s reputation in the community? Why do you think this is? |
| Appropriateness | Do you think the customers at your shop like that HIVST is available at your shop? (***Probe about AGYW.***) Why or why not?  Which kinds of customers are most interested in HIVST? Why do you think this is?  Do you feel that your customers are able to correctly use HIVST after obtaining it from your shop? (***Probe about AGYW.***) Why do you think this?   - Can you tell me about a customer who you felt would not be able to use HIVST correctly on his/her own? What did you do?   Can you describe how providing HIVST has impacted the work flow or daily operations of your shop? Why do you think this? |
| Challenges/ adverse events | To your knowledge, have any customers had negative experiences as a result of your shop offering HIVST? Tell me about these.  Tell me about any other challenges you experienced in offering HIVST to your customers. (***Probe about AGYW.***) |
| Sales and pricing | Are you satisfied with sales over the past few months? Why/why not?  What pricing strategies did you employ?   - E.g. Bundling with other products, promotions, sales targets, etc.   How do you decide how to price HIVST? ***Probe about the following topics****:*   - What factors do shop owners consider when deciding how to price HIVST? - Are they affected by prices of other shops? How much information do shop owners have about others? To what extent do they interact/compete/collude on price? - How do stocking and cash flow considerations affect pricing? - How do profit margins (in absolute or relative terms) affect pricing decisions? - Do pro-social motivations affect pricing strategy? |
| Sustainability | Would you like to continue to offer HIVST at your shop on a long-term basis? Why or why not? (***Probe about acceptable procurement costs.***)   - ***If no:*** Is there anything that might motivate you to continue to offer HIVST on a long-term basis?   To what extent do you think offering HIVST at drug shops like yours on a long-term basis would encourage people to test regularly? (***Probe about AGYW.***)  What do you foresee as the major barriers to continuing to offer HIVST in your shop? (***Probe about resources they may be lacking.***)  What are the facilitators? (***Probe about resources they*** ***have available to help with scale up and maintenance*.**)  Do you have any other feedback or suggestions about offering HIVST at ADDOs? |

**D. Intervention ADDOs**

Next, I will ask you about your experience with the Queen Club program, which includes the loyalty and symbol card and mystery boxes.

Please keep in mind that there are no right or wrong answers to these questions. We are interested in understanding how you and your customers feel about this program and about having this program in your shop.

| **Topic** | **Main question** |
| --- | --- |
| Adoption | Have you been using this program with some of the customers you serve?   - Why or why not? - Which parts of the program have you been using? |
| Fidelity  Appropriateness | Please describe how you use the program and how it works in your shop.  You may have helped to recruit girls for the program while working at the shop. Can you tell me about the last time you recruited a girl for the program?   - How did you identify her as a potentially eligible customer? - How did you discuss the program with her? What did you say? - Does recruiting for the program impact your work flow? How? - Have you encountered any challenges while recruiting girls into the program? Have you ever had a girl decline to join the program? Tell me about what happened. - What works well while recruiting customers into the program? What works less well? Can you give me a specific example?   Customers in the program were able to earn stamps on a loyalty card. Can you tell me about the last time a customer used the loyalty card in your shop to earn a stamp? Please describe the process from when she entered your shop to when she left.   - Has anything about the loyalty card process been difficult to implement or confusing for you, your staff, or your customers? What/why? - What would you change about the loyalty card process? Why? (***Probe about conditions for earning stamps.***) - Have you experienced any benefits from offering loyalty cards? (***Probe about repeat customers, business.***)   Customers were able to earn draws from a mystery box. You may have administered this component of the program while working at the shop. Please describe the last time a customer earned a draw from the mystery box.   - How did she react to the mystery boxes? The product that she drew? - How did you feel while allowing girls to draw from the mystery box? - In general, which products did girls seem most or least excited to draw? - Did you encounter any challenges while integrating this component of the program into your work at the shop? - What would you change about the mystery boxes? Why? (***Probe about products in the boxes.***)   During the program, customers could point to symbols on the back of the loyalty card to request certain products. Can you tell me about the last time a customer did this?   - What did she do or say? Which product did she request? What did you do or say? Did she receive the product? For what cost? - Did you encounter any challenges while integrating this component of the program into your work at the shop? (***Probe about customers understanding how to use symbol cards.***) - What would you change about the symbol card? *(****Probe about symbols, adding or removing products.***)   Tell me about the last time a customer interacted with the product display and tablet videos. (***Probe about how and with which products/videos she interacted.***)   - Did you encounter any challenges while integrating this component of the program into your work? (***Probe about customers lingering in shop*.**)   ***ADDO owner only*:** How confident are you that your staff have been offering the program as it was intended to be offered? |
| Acceptability | What did you think about the program?   - What do you like about it? Dislike about it? - What has been easy about it? Difficult about it?   How do your colleagues in the shop generally feel about the program? What kinds of things do they say about the program?  Do you think the program has been helpful to you in doing your job, or has it made your job harder? Why?   - Can you tell me about a time that the program helped you better serve a customer? - Please tell me about a time when the program made your job harder.   How do you think the program has affected business in the shop? Why do you think this is?   - Have you experienced any changes in how many or the types of customers who come to your shop? (***Probe about AGYW, repeat customers*.**) Why do you think this is? - Have you experienced any changes in how many sales you are making? Why do you think this is? Which products account for this?   How has the program affected the shop’s reputation in the community? Why do you think this is? |
| Appropriateness | Which parts of the program did your customers like or use most? Dislike or use least?  What parts of the program should be kept? Which parts have worked well? Why?  What parts should be removed or changed? What didn’t work well? Why?  Can you describe how the program has changed the work environment or operations in your shop? Think back to before the program, maybe in May or June. Is your daily work any different from then? Why do you think this?  To your knowledge, have any customers had negative experiences as a result of your shop offering this program? Tell me about these.  What have customers who were ***not*** eligible for the program said about it? |
| Sustainability | Would you like to continue to offer this program at your shop on a long-term basis? Why or why not?   - ***If no:*** How might the program be changed to motivate you to continue to integrate the program into your work on a long-term basis? - ***If yes:*** Under what conditions would you continue to offer this program? ***Probe about paying for supplies.***   To what extent do you think a program like this would encourage girls to test regularly on a long-term basis? To use contraception? Why/?  What do you foresee as the major barriers to sustained use of this program in your shop? (***Probe about resources they may be lacking.***)  What are the facilitators? (***Probe about resources they*** ***have available to help with scale up and maintenance*.**)  Do you have any other feedback/ suggestions about the program? |

Last, I have a few questions about the program which you can answer privately on your own. For each question, please choose whether you strongly agree to strongly disagree, or somewhere in between. Please let me know if you would like for me to read or explain any question.

|  | Strongly agree | Agree | Neither agree nor disagree | Disagree | Strongly disagree |
| --- | --- | --- | --- | --- | --- |
| I think that I would like to continue to offer this program. |  |  |  |  |  |
| I found the program unnecessarily complex. |  |  |  |  |  |
| I thought the program was easy to offer in my shop. |  |  |  |  |  |
| I think that I would need the support of a technical person to be able to continue this program. |  |  |  |  |  |
| I found the various components of the program to be well-integrated. |  |  |  |  |  |
| I thought that this program was too complicated. |  |  |  |  |  |
| I think that most people would learn to offer this program very quickly. |  |  |  |  |  |
| I found the program inconvenient to offer. |  |  |  |  |  |
| I felt very confident offering the program. |  |  |  |  |  |
| I needed to learn a lot of things before I could start offering this program. |  |  |  |  |  |

**E. Comparison ADDOs**

***Describe the intervention (showing the card and mystery box) and summarize the experiences of the intervention from in-depth interviews.***

Would you like to offer this program at your shop? Why or why not? Which components?

- ***If no:*** How might the program be changed to motivate you to continue to integrate the program into your work on a long-term basis?
- ***If yes:*** Under what conditions would you offer this program at your shop on a long-term basis? ***Probe about paying for supplies.***

What would you change about the program? (***Probe about each component.***)

***Turn off recorder, thank the respondent for his/her time, and give him/her the 15000 TSH.***
