# Supplementary material for: “They call me the ‘Great Queen’”: implementing the Malkia Klabu program to improve access to HIV self-testing and contraception for adolescent girls and young women in Tanzania
Source: Reprod Health. 2024 Feb 7;21:21. doi: 10.1186/s12978-024-01744-x (PMC10848389; doi:10.1186/s12978-024-01744-x)
Supplement: Supplementary file 2 — Additional file 2: In-Depth Interview Guide—Adolescent Girls and Young Women. [file 12978_2024_1744_MOESM2_ESM.docx]

# In-Depth Interview Guide – Adolescent Girls and Young Women

_________________________________________________________________________

|  | Date: | **(DD/MM/YY)**: ____ /____ / ____ |
| --- | --- | --- |
|  | Interviewer name: |  |
|  | Participant ID: |  |
|  | Participant age: |  |
| 5. | Start time: |  |
| 6. | Finish time: |  |

**Introduction and consent**

1. ***Introduce yourself and the study.***
2. ***Obtain written informed consent or assent [READ CONSENT FORM]***
3. Did you ask if the participant has any questions?  YES  NO
4. Did participant agree to participate?  YES

NO 🡪 *STOP*

1. Did you give participant a copy of consent?  YES  NO
2. ***Obtain permission to audio record***

We would like to audio-record the interview so that we can remember everything we discuss here today.

1. Is it okay with you if I audio-record?  YES 🡪 *TURN ON RECORDER*

NO 🡪 *TAKE NOTES*

1. ***Turn on recorder and say Participant ID into the recorder!***

***Instructions:*** ***The questions below outline main categories of questioning. The sub bullets detail topics for further probing. There is no need to ask every question, but rather to focus on the areas in which the respondent has the most to say.***

Before we start, I would like to remind you of some important things to keep in mind during our conversation. Please remember that everything about this study is completely voluntary, and you should not feel compelled to share anything you do not want to. Everything you say will be kept confidential; nothing will be shared with parents, teachers, or anyone else outside of the research team. As we are asking questions that might lead you to think about sensitive topics, if you do not like a question, or if you want to leave before the interview is over, you are free to do so. Nothing bad will happen.

**A. Introduction**

First, I’d like to get to know you a little better.

Can you tell me a bit about yourself? (***To ease into the interview, ask the participant about some basic information, such as what she does for fun, how many people she has in her family, her school grade and/or what she studies, etc.****)*

What are some of the things that are going well for you in your life right now that make you happy?

What are some of the things that are more difficult?

**B. Drug shop experiences**

I would like to ask you some questions about your recent experiences visiting a drug shop.

Tell me about the last time you visited [***name of ADDO***].

- Why did you choose to go to that drug shop instead of another place?
- What did you want at the shop? What items did you actually buy or receive? How come? (***Probe specifically about HIV self-test and contraception.***)
- About how much money did you spend?
- Did the shopkeeper provide you with a referral to any health services? (***Probe specifically about HIV testing/treatment and contraception.***)
- Did you encounter any challenges in getting what you wanted? (***Probe***: Where did you get the money?)
- What time of day did you go? Why did you go then?
- Who was with you? Why?
- Were there other customers in the shop while you were there?
  - How did they make you feel? Did they affect anything you wanted to do?
- Is this similar to other times in the last month that you’ve visited a drug shop?
  - How many times have you visited in the last month? The last two weeks?

Tell me about your interaction with the people who work there during the visit when we met you.

- Who attended to you? What were they like?
- How familiar are they with you? With your family or friends?
- Was there anything that you liked about that shop keeper? Anything you didn’t like? (Remember that I won’t tell anything you say to anyone who works at the shop.)
- What did they say to you? How did they treat you?
- How did this affect your decision to buy things there or not?

What were you feeling before you went there? While you were there? And after you left?

- Did you feel comfortable at the shop? Why or why not?

***If the participant sought and/or received HIVST*:**

What led you to decide to pick up the test? How did you first learn about it?

- How did you decide where to go to get this? How did you know this was available there?
- Did you pick up the test for yourself or someone else? (***If someone else, probe about who, why they picked it up for this person, and whether they sold it.***)
- Did you encounter any challenges in getting what you needed?
- Did you feel comfortable asking the shop keeper for this product? Why or why not?
- Did you feel you had enough information on how to do the test properly? (***Probe about counseling, instructions, video.***)

Tell me about your experiences taking the test.

- When and where did you take the test? Why?
- Did anyone help you? Why or why not?
- How did you feel before you took the test? While you took the test?
- Tell me about learning the results.
  - How did you feel?
  - Did you trust the results?
  - Did you repeat the test?
- What did you do with the test after you took it? Why?
- Were you happy that you took the test, or did you regret taking the test? Why?
- Was this your first time testing for HIV? ***If no:*** Can you tell me how it compared to your previous experience?

After you received the test, did you try to contact anyone with questions about the test or to get follow-up care? (***Probe about whether they engaged with the ADDO seller or HIV referral staff.***)

- Who did you contact? Why did you contact them?
- Tell me about your interaction with this person. How did they treat you?
- What did they say to you? Did they [***answer your questions / link you to confirmatory testing or care***]?
- Did you face any challenges in having your needs met?

Have you had any negative experiences as a result of receiving this test from the ADDO? Tell me about these.

Would you get this test again? Would you recommend it to friends? Why or why not?

***If participant did not seek or receive HIVST:***

Were you aware that this shop offers free HIV self-test kits for adolescent girls and young women? ***If yes:***

- How did you know they were available there?
- Have you ever received one from this drug shop or another drug shop?
  - ***If yes, ask questions about experience in section above.***
  - ***If no*:** Can you tell me about why you did not receive this product? (***Probe about interest in product and challenges in obtaining it.***)

***If participant sought and/or received contraception:***

Can you tell me about why you decided to get this method?

- How did you decide where to go? How did you know they had what you were looking for?
- Have you used this method before? ***If yes:*** Where did you get it last time?
- Why did you choose this method? Tell me about how you learned about it.
- Was it difficult to get that product or easy? Did you encounter any challenges in getting what you needed?
- Were you comfortable asking the shop keeper for this product? Why or why not? How did they respond?
- Did you feel you had enough information on how to use this method properly? (***Probe about counseling, instructions, video.***)
- How does your partner (or partners) feel about you using this method?

***If participant received a referral:***

Can you tell me about how the shopkeeper referred you for this service?

Did you visit the facility or health provider to which the shopkeeper referred you?

- ***If no:*** Why not? Tell me about any challenges you experienced.
- ***If yes:*** Walk me through your visit to the facility. With whom did you interact, and what was your experience like?
  - Did you receive the service that you were seeking?
    - ***If no:*** Why not?
    - ***If seeking contraception:*** Did you get a contraceptive product? How did you decide which contraceptive method to get?
  - Were you satisfied with your visit?
  - What was the best part of the visit? The worst part?

**C. Intervention exposure**

Finally, I would like to ask you about a program offered in the community. Have you ever heard of a program called the “Queen Club”?

- ***If yes:*** Can you tell me what you have heard about it?

***Regardless of response, show the Queen Card.***

Have you ever seen this before?

- ***If yes:*** When did you see it? Can you tell me anything about what it is for?

***If familiar with program or card:***

We are interested in understanding how you felt about this program. Please keep in mind that there are no right or wrong answers to these questions.

| **Topic** | **Question** |
| --- | --- |
| Adoption  Fidelity | Can you tell me about what you know about the program? (***Probe about loyalty/symbol card and mystery boxes, free products, and display/tablet if needed.***)  How did you learn about this program?   - ***If at ADDO:*** Tell me about your interaction with the shop keeper when you heard about the program.   Did you find anything confusing about it? Exciting about it?  Did you participate in this program? Why or why not?   - How long have you been a member? Which parts did you participate in? - ***If no:*** How might the program be changed to motivate you to join? (***Probe about each program component.***) - Was it easy or challenging to sign up? Tell me about your experience signing up. (***Probe about videos.***) - Did you participate in the program at one drug shop? At more than one? |
| Acceptability  Appropriateness | How has your experience been participating in the program?   - Can you tell me some good things about it and some not so good things about it? - Are there specific parts of the program you like more than others? Why?   Do you think that this program has been helpful to you? Why or why not?  Have you had any negative experiences as a result of your participation in this program? Tell me about these.  Have you talked with anyone else about this program? Who? What did you say? (***Probe about family, friends, partners.***)   - Who knew that you were participating in this program? |
| Loyalty card/ mystery boxes | Can you describe your experience using the Queen Club card to earn stamps?   - How many stamps do you think you’ve earned? (***Ask to see card, if she has it.***) How many cards have you had? - Where do you keep the card? Why?   Were there any challenges that made it hard for you to use the card or to earn stamps? (***Probe about having time and permission to go to the ADDO and having money to purchase something.***)  Can you tell me about the last time you earned a stamp?   - Why did you go to the drug shop that day? - What did you purchase to earn the stamp?   Can you tell me about the last time you drew an item from a mystery box?   - How did you feel about what you drew from the mystery box? - How did you use what you drew? - Was this your first time drawing from a mystery box? ***If no:*** How did this compare to the previous time? - Are there any other items that you or other girls would be excited to receive in a mystery box? |
| Symbol card/ contraception | Did you use the symbols on the back of the card to try to obtain any of these products? Why or why not? Which products?  ***If yes:*** Describe the last time you used the symbol card to try to obtain a product.   - What did you do? How did the shop keeper respond? - Did you experience any challenges in getting what you wanted? (***Probe about perceptions of privacy and judgement, whether item was free.***)   Are there times where the symbol card did not work well? Tell me about a time when you were frustrated with the symbol card.  Did you look at the display of sexual health products, like contraception? ***If yes:*** What did you think about this experience? Tell me about what you liked and disliked.  Did you watch any videos about contraception on the tablet? ***If yes:*** What did you think about this experience? Tell me about what you liked and disliked. |
| Sustainability | Would you like to continue to be a member of this program on a long-term basis? Why or why not?   - ***If no:*** How might the program be changed to motivate you to continue to use the program?   What do you think are the major challenges to your continued use of the program?  Is there anything in your life that makes it easier for you to continue to use the program?  Do you have any other feedback or suggestions about how the program could be improved to better serve girls/women your age? |

***If not familiar with program or card:***

***Briefly explain the program (loyalty/symbol card, mystery boxes, free products, display/tablet, etc.).***

Would you sign up for this program, if offered? Why or why not?

- ***If no:*** Do you have any suggestions about how the program could be changed to motivate you to sign up for the program?
- ***Probe about opinions on different components of the program.***

***Turn off recorder, thank the respondent for her time, and give her the 10,000 TSH.***
