# Supplementary material for: “They call me the ‘Great Queen’”: implementing the Malkia Klabu program to improve access to HIV self-testing and contraception for adolescent girls and young women in Tanzania
Source: Reprod Health. 2024 Feb 7;21:21. doi: 10.1186/s12978-024-01744-x (PMC10848389; doi:10.1186/s12978-024-01744-x)
Supplement: Supplementary file 3 — Additional file 3: In-Depth Interview Guide—Health Facility Staff. [file 12978_2024_1744_MOESM3_ESM.docx]

# In-Depth Interview Guide – Health Facility Staff

_________________________________________________________________________

|  | Date: | **(DD/MM/YY)**: ____ /____ / ____ |
| --- | --- | --- |
|  | Interviewer name: |  |
|  | Participant ID: |  |
|  | Participant age: |  |
|  | Participant sex: |  |
|  | Participant role at facility: |  |
| 7. | Start time: |  |
| 8. | Finish time: |  |

**Introduction and consent**

1. ***Introduce yourself and the study.***
2. ***Obtain written informed consent [READ CONSENT FORM]***
3. Did you ask if the participant has any questions?  YES  NO
4. Did participant agree to participate?  YES

NO 🡪 *STOP*

1. Did you give participant a copy of consent?  YES  NO
2. ***Obtain permission to audio record***

We would like to audio-record the interview so that we can record everything we discuss here today. You can also request that I turn off the recorder at any time.

1. Is it okay with you if I audio-record?  YES 🡪 *TURN ON RECORDER*

NO 🡪 *TAKE NOTES*

1. ***Turn on recorder and say Participant ID into the recorder!***

***Instructions: The questions below outline main categories of questioning. The sub bullets detail topics for further probing. There is no need to ask every question, but rather to focus on the areas in which the respondent has the most to say.***

Before we start, I would like to remind you of some important things to keep in mind during our conversation. Please remember that everything about this study is completely voluntary, and you should not feel compelled to share anything you do not want to. Everything you say will be kept confidential; nothing will be shared with government officials, other people who work at your facility, or anyone outside of the research team. As we are asking questions that might lead you to think about sensitive topics, if you do not like a question, or if you want to end the interview, you are free to do so. Nothing bad will happen.

Some of the questions ask about clients who contacted you by phone or visited your facility. When you are answering these questions, please don’t tell me the clients’ names or identifying information about them.

Some questions ask about adolescent girls and young women in your community. When I say “adolescent girls and young women” (or simply “girls”), I am talking about people around ages 15 to 24 years old.

**A. Introduction**

First, I’d like to learn a little bit about you and your time working at this health facility:

How long have you worked at this health facility? Why did you start working here?

What’s the best part about your work? What about it do you enjoy or makes you happy?

What’s the hardest part about your work?

**B. HIV self-testing referral experiences**

Now I will ask you about your experiences counseling people who received HIV self-test kits from drug shops participating in our study.

| **Construct** | **Question** |
| --- | --- |
| Adoption  Fidelity | Can you describe how often you were contacted by people who received HIV self-test kits during the study period?   - Tell me about the types of people who called. (***Probe about whether people other than adolescent girls and young women called.***) - In what ways did people usually contact you? (***Probe about calls, SMS, in-person.***) - How often were you able to speak with people who called? (***Probe about missed calls, voicemails, replying to SMS, etc. and any challenges with keeping up with calls.***)   What were the most frequent questions or needs of the clients who contacted you?   - ***Probe about interpreting test results and need for confirmatory testing, if not mentioned.*** - ***If a specific question about HIV self-testing is mentioned, probe about how s/he responded.***   Can you tell me about the last time that an adolescent girl or young woman contacted you because she thought she had a positive (reactive) HIV self-test?   - What did she say? Can you describe her reaction to this result? - How did her reaction compare to the reactions of girls that you see after positive tests in the clinic? - Can you tell me what you said? How did she respond? - Do you know what she did after speaking with you? (***Probe about confirmatory testing and linkage to care.***)   Were any adolescent girls or young woman that contacted you able to receive confirmatory testing and/or linked to care? ***If yes:*** Please tell me about what happened.  Can you tell me about the last time that an adolescent girl or young woman contacted you because she had a question about how to use or interpret the HIV self-test kit?   - What did she say? How did you respond? - Do you know what she did after speaking with you? |
| Acceptability  Appropriateness | Did you feel that you were able to counsel clients appropriately over the phone?   - What aspects of counseling clients over the phone were easy? Hard? How did it compare to counseling clients in the clinic? - What worked particularly well? What worked less well? (***Probe for specific examples.***)   Do you think that making HIV self-test kits available in this community has been helpful to you in doing your job, or has it made your job harder? Why? (***Probe for specific examples.***)  Did you encounter any challenges while answering clients’ questions about HIV self-testing? While linking clients to confirmatory testing or HIV treatment? (***Probe for specific examples.***)  In your role, were you aware of any negative experiences that were associated with HIV self-testing? Tell me about these.  Has distributing HIV self-test kits in the community changed the work environment in your clinic? How? Why do you think this? |
| Sustainability | What is your opinion about whether we should continue to distribute HIV self-test kits in local drug shops? Why?  Suppose I offered you the opportunity to continue to integrate HIV self-testing into your counseling sessions on a long-term basis – would you agree? Why or why?   - ***If no:*** How could the program be changed to motivate you to continue to counsel clients on HIV self-testing on a long-term basis?   To what extent do you think distributing HIV self-test kits would encourage clients to test regularly on a long-term basis? Why?  What do you foresee as the major barriers to expanding access to HIV self-testing in the community?  What do you see as the major barriers to linking people who use HIV self-test kits to confirmatory testing and treatment?  Describe what resources you have available in your facility to help with scale up and maintenance of confirmatory testing and linkage to care for clients who use HIV self-test kits.  Are there any resources you are lacking?  Do you have any other feedback or suggestions about how to best to distribute HIV self-test kits in drug shops in order to enable people who self-test to link to confirmatory testing and treatment? |

**C. Contraception Referral Experiences**

| **Construct** | **Question** |
| --- | --- |
| Adoption  Fidelity | Can you describe how often you were contacted by people who were seeking contraception during the study period?   - Tell me about the types of people who called. (***Probe about whether people other than adolescent girls and young women called.***) - What were the most frequent questions or needs of the clients who contacted you? ***Probe about methods sought.*** - ***If a specific question or request for contraception is mentioned, probe about how s/he responded.***   Can you tell me about the last time that an adolescent girl or young woman contacted you because she had a question about family planning or contraception?   - What did she say? How did you respond? - Do you know what she did after speaking with you?   Were any adolescent girls or young woman that contacted you able to receive a method at a health facility? ***If yes:*** Please tell me about what happened. |
| Acceptability  Appropriateness | Did you encounter any challenges while counseling clients who were seeking contraception? (***Probe for specific examples.***)  In your role, were you aware of any negative experiences that were associated with contraception? Tell me about these. |
| Sustainability | What is your opinion about whether we should continue to link girls to contraceptive services through local drug shops? Why?  Suppose I offered you the opportunity to continue to counsel girls about contraception on a long-term basis – would you agree? Why or why?   - ***If no:*** How could the program be changed to motivate you to continue to counsel clients on contraception on a long-term basis?   What do you foresee as the major barriers to expanding access to contraception to girls in the community?  Do you have any other feedback or suggestions about how to help girls in the community access contraception? |

**D. Wrapping up**

How was your experience collaborating with Health for a Prosperous Nation on this research project?

- What were the best parts? The most challenging parts?
- Was there anything that would have made this collaboration go more smoothly?

Do you have any advice or feedback on how we should engage with HIV counselors, such as yourself, in future projects?

***Turn off recorder, thank the respondent for his/her time, and give him/her the 15,000 TSH.***
